# Quarterly Report (No. 5,) of the Medical Cases Treated at the Westminster General Dispensary, from February 10th to May 10th, 1823

**Published:** 1823-06

**Authors:** R. Macleod

**Affiliations:** one of the Physicians to that Institution.


### STATISTICAL MEDICINE.

ART. I.—*Quarterly Report (No. 5,) of the Medical Cases treated at the Westminster General Dispensary, from February 10th to May 10th*, 1823. By R. MACLEOD, M.D. one of the Physicians to that Institution.

*Diseases affecting particular Organs.*

| Organs | Division | Disease | Form | Cases | Total |
|---|---|---|---|---|---|
| Head and Nervous System | | Apoplexy | | 1 | |
| | | Hydrocephalus Acutus | | 1 | |
| | | Determination to the Head | | 11 | |
| | | Hemiplegia | | 3 | |
| | | Epilepsy | | 5 | |
| | | Hysteria | | 13 | |
| | | Hypochondriasis | | 2 | |
| | | Chorea | | 3 | = 39 |
| Nostrils, Fauces, Mouth, and Throat | | Stricture of the Œsophagus | | 1 | |
| | | Epistaxis | | 2 | |
| | | Catarrh | | 22 | |
| | | Cynanche | Tonsillaris | 4 | |
| | | | Parotidea | 2 | |
| | | Chronic Disease of the Larynx | | 3 | = 34 |
| Organs of Respiration | | Dyspnœa | | 1 | |
| | | Bronchitis | Acute | 4 | |
| | | | Chronic | 39 | |
| | | Pneumonia | Acute | 2 | |
| | | | Subacute | 11 | |
| | | Hæmoptisis | | 4 | |
| | | Phthisis, in various stages | | 25 | = 86 |
| Organs of Digestion | Stomach | Dyspepsia (in various forms) | | 44 | |
| | Bowels | Constipation | | 8 | |
| | | Colic | | 1 | |
| | | Diarrhœa | | 4 | |
| | | Dysentery | | 1 | |
| | | Irritation from | Tape-worm | 1 | |
| | | | Lumbricus | 1 | |
| | | | Ascarides | 2 | |
| | | Tympanitis | | 1 | |
| | Liver | Hepatitis, Chronic | | 11 | |
| | | Organic Disease of Liver | | 1 | |
| | | Icterus | | 1 | = 67 |
| Effusion into the Abdominal Cavity, from Rupture of the Alimentary Canal | | | | | 1 |
| Organs of Urine | | Nephralgia | | | 2 |
| Organs of Generation | | Amenorrhœa | | 2 | |
| | | Menorrhagia | | 2 | |
| | | Leucorrhœa | | 5 | = 9 |
| Skin | | Erysipelas | | 1 | |
| | | Urticaria | | 1 | |
| | | Psoriasis | | 1 | = 3 |
| Muscles, Tendons, Joints, &c. | | Gout | | 1 | |
| | | Rheumatism | Acute | 9 | |
| | | | Chronic | 17 | = 27 |

*Diseases not easily referred to particular Organs.*

| Disease | Form | Cases | Total |
|---|---|---|---|
| Fevers | Remittent | 1 | |
| | Continued | 12 | = 13 |

| | | | |
|---|---|---|---|
| Dropsies | Hydrothorax | 3 | |
| | Ascites | 3 | |
| | General Dropsy | 1 = | 7 |
| | Total | | 288 |

Fatal Cases:—Apoplexy, 1; Hydrocephalus, 1; Hydrothorax, 2; General Dropsy, 1; Phthisis, 3; Disease of Liver, 1; Effusion into the Cavity of the Abdomen, 1, = 10.

The diseases prevalent during the last three months have been more severe than is usual at this season, and the majority still consists of affections of the organs of respiration, although not so remarkably as during the three months preceding.

In one of the cases of chronic disease of the larynx, the tar-vapour, recommended by Sir A. Crichton as peculiarly applicable to this form of disease, has been tried; but without any apparent benefit. It is proper, however, to remark, that it was used through an inhaler, as it was found impossible to impregnate the apartment with the vapour to a sufficient extent, and because the lungs, appearing nearly sound, were not fatigued with the exertion.

A case of very severe pain of the brow, putting on the characters of tic douloureux, which resisted topical bleeding, purging, &c. for three weeks, was relieved in as many days by large doses of the subcarbonate of iron, and in a few more apparently cured, as the patient has not returned for a month.

Of the fatal cases, that from effusion of the alimentary matters into the cavity of the peritoneum is the only one which merits particular description.—I was sent for, at three o'clock one afternoon, (last month,) to visit a labourer, about fifty years of age, whom I found under the following circumstances: He was lying on the bed, with his clothes on; but raised himself on my entrance, and described the particulars of his attack. He had been taken ill (he said) about three o'clock in the morning with violent pain in the lower part of the belly, accompanied with great faintness, having taken a large dose of castor-oil (about an ounce) the night before: he had been habitually subject to constipation, and difficulty of making water. His countenance was expressive of mortal anxiety; his extremities cold; and his pulse frequent, and very feeble. He died at six o'clock the same afternoon, having survived the first attack fifteen hours. The brain and contents of the chest were sound. On opening the abdomen, about two pints and a half of thin feculent matter were found in the peritoneal sac. The bowels had a general blush of redness; and the caput cœcum coli, together with the cœcum and colon in the immediate vicinity, were of a deep purple, and glued to the neighbouring parts by new bands of adhesion. Examination, as careful as the circumstances admitted of, did not enable us to detect the exact situation of the ruptured or ulcerated portion, although it was probably situated about these parts.—The reader will find some interesting cases of this nature, with remarks by the Editor, in the Number of the "Reveu Medicale" for February.

